# Periodic Ophthalmia1Read before the meeting of the Illinois Veterinary Medical and Surgical Association.

**Published:** 1900-01

**Authors:** I. M. Luzader

**Affiliations:** Nokomis, Ill.


### PERIODIC OPHTHALMIA.[1]

By I. M. Luzader, V.S.,
NOKOMIS, ILL.

This affection is intimately related to certain climates, systems, and soils, and shows a strong tendency to occur again, and usually terminates in blindness from cataract.

Its causes may be said to be primarily in the soil of frequently submerged ground.

Heredity is one of the most potent causes. We know this is very positively demonstrated when both parents have suffered. In support of this we know if a mare had borne a number of foals, all sound, and then suffered an attack of periodic ophthalmia the subsequently born would also suffer. The study of atavism presents many interesting facts in these matters. Unwholesome food and error in feed are undoubtedly predisposing causes.

The symptoms of the disease are those which usually come on very suddenly ; perhaps in a single night, a great tenderness in one eye, commonly marked by the eyelids being shut ; a copious secretion of tears ; the white of the whole anterior chamber dim and clouded, there being no distinct speck on the cornea ; the redness of the eyeball is never very remarkable, even when the disease assumes its most aggravated form ; the dimness of the anterior chamber increases rapidly, and in two or three days, or even less time, a yellow spot usually appears at the bottom of that cavity, arising from a formation of pus ; after lasting from seven to twenty-one days the inflammation and watering usually begins to gradually subside, and absorption usually requires from twelve to fifteen days.

The recurrence is the characteristic of the affection, and it will recur again and again until total loss of sight ensues.

These recurrences are determined more likely by some periodicity of the system, from five to eight attacks usually suffice in resulting blindness.

After the first attack there can usually be seen a bluish ring around the corneal margin, the eye, therefore, seeming smaller, and after several attacks it is smaller from atrophy.

Now this is a general picture, but that the attacks vary with different cases must be remembered.

[1] Read before the meeting of the Illinois Veterinary Medical and Surgical Association.

The recurrence, however, is characteristic, and alike leads to cataract and blindness.

The prevention of this disease is the great object, and to accomplish this most desirable end we must go back to the starting-point, and have careful and discriminating breeding, feeding, stabling, etc.

*Treatment.* Some are benefited by colchicum in scruple doses, where rheumatic tendencies are evinced, or two-drachm doses of salicylate of soda twice daily. When convalescing, tonics of nux vomica, gr. x.; sulphate of soda, ʒj., daily.

